# Missed opportunities for immunization among children 0 to 11 months of age that were attended to at debre tabor comprehensive specialized hospital, south gondar zone, Ethiopia

**DOI:** 10.3389/fped.2023.1169328

**Published:** 2023-04-27

**Authors:** Bekalu Getnet Kassa, Nhial Char Lul

**Affiliations:** Department of Midwifery, College of Health Science, Debre Tabor University, Debre Tabor, Ethiopia

**Keywords:** missed opportunities, immunization, vaccine, dropout, Ethiopia

## Abstract

**Background:**

The World Health Organization's Expanded Immunization Program was established in 1974 and aimed to provide vaccines to children all over the world. Since the inception of this program, numerous initiatives and campaigns have been launched, and millions of children around the world have been saved from death. Many vaccine-preventable diseases, however, remain prevalent in developing countries. This is because most of those countries have low immunization coverage for an unknown number of reasons. As a result, the goal of this study was to examine missed opportunities for immunization among children aged 0 to 11 months.

**Methods:**

A cross-sectional survey was carried out from May to August 2022. A structured questionnaire was used to collect data, and the sample was chosen using a simple random sampling technique. Before being entered into the Epidata and exported to the Statistical Package for Social Science for analysis, the data were checked for consistency and completeness. The statistical significance was determined using binary and multiple logistic regression analyses. The statistical level of significance was established at *p* ≤ 0.05.

**Result:**

In this study, 49.1% of immunization opportunities were missed. Education status [AOR = 2.45, 95% CI = 2.14, 4.22], rural residence [AOR = 4.32, 95% CI = 3.11, 6.38], and perception of caretakers [AOR = 2.13, 95% CI = 1.89, 4.07] were associated with the missed opportunity of immunization.

**Conclusion:**

When compared to previous studies, the proportion of missed immunization opportunities was high in this study. The healthcare staff should be applying the multi-dose vial policy, which is recommended by the World Health Organization to increase the services. The doses for BCG and measles should be minimized to lower doses per vial in order to conduct immunization without having to wait for enough children and without worrying about vaccine waste. All infants who visit the hospital should be linked to immunization services.

## Background

By the age of 9 to 12 months, children in Ethiopia are considered completely vaccinated if they have obtained one dose of Bacillus Calmette Guerin (BCG), three doses of DPT, three doses of polio vaccines, and one dose of measles vaccination (1, 2). A missed opportunity for vaccination (MOV) refers to any contact with health services by an individual (child or person of any age) who is eligible for vaccination (e.g., unvaccinated or partially vaccinated and free of contraindications to vaccination), which does not result in the person receiving one or more of the vaccine doses for which he or she is eligible (3–5).

Diseases covered by the Expanded Program on Immunization (EPI) are highly contagious and account for more than half of all child morbidity and mortality (6). Every year, over nineteen million children miss out on the benefits of complete vaccination, and many receive no vaccines at all, which results in >1 million deaths each year. Nearly 30% of deaths among under-5-year-old children are caused by vaccine-preventable diseases (7).

An increase in global immunization coverage would save 1.5 million lives (8). Despite a significant increase in immunization services in Africa, many children remain unvaccinated or under-vaccinated (9). Routine immunization performance in the African Region has stalled for the majority of vaccine-delivered antigens over the last decade.

The country with the second-highest proportion of unvaccinated children, after Nigeria, is Ethiopia, where vaccine coverage was reported to be 75% in 2020 (3). To minimize such problems, the World Health Organization recommended a multi-dose vial policy across each country to fulfill the criteria of the multi-dose vial policy (10). In absolute figures, the majority of deaths are still attributable to a small number of conditions that can be avoided using existing interventions through vaccinations (11).

Ethiopia, one of the ten nations with the highest global non-vaccination prevalence, is below the WHO target of vaccine coverage. Morbidity and mortality from diseases that can be prevented by vaccination are still high, with diarrheal illnesses, respiratory infections, and tuberculosis ranking among the top five killers of young children (12). The infant mortality rate stood at 47, and the mortality of under 5-year-old children was 59 per thousand live births in Ethiopia (1). Vaccination, the most cost-effective measure for public health, has protected children around the world from common illnesses (13). Several factors have been identified as contributing to missed opportunities such as antenatal care visits, residence, educational status, high fertility rates, place of delivery, perceived health care support, wealth index, occupational status, religious affiliation, mothers’ awareness of vaccination, and parity (5, 7, 14–16). As a result, further studies are required to determine the main factors for missed opportunities for immunization.

## Methods

### Study area

The study was conducted at the Debre Tabor Comprehensive and Specialized Hospital located in Debre Tabor town, south Gondar zone, Amhara National Regional State, Ethiopia, which is 667 kilometers north of Addis Ababa, the capital city of Ethiopia. The hospital has been offering preventive, delivery, and curative healthcare services to approximately 2.7 million people. It serves as a teaching hospital for Debre Tabor University's College of Health Sciences.

### Study design and period

A facility-based cross-sectional study was carried out from May to August 2022.

### Population

All infants aged between 0 and 11 months were the source populations. Infants aged 0 to 11 months who visited Debre Tabor Comprehensive Specialized Hospital during the study period were the sampled population. All infants aged 0 to 11 months who were seriously ill or hospitalized were excluded.

### Sample size determination and sampling procedures

The required sample size for this study was determined using the following assumptions: desired precision (d) = 5%, confidence level = 95% (Z*α*/2 = ±1.96 value), and prevalence of missed opportunity of 28.8% (17). As a result, with a 10% non-response rate, the final sample size was 346. A simple random sampling technique was employed to select 346 study participants who fulfilled the inclusion criteria from the source population. The sample size was then determined by drawing a sample randomly by the lottery method from each visit until the desired sample size was gotten.

### Study variables

**Dependent variable:** Missed opportunity for vaccination (Yes/No).

**Independent variable:** Maternal educational status, false contraindication for vaccination, health workers' practice and knowledge, side effects of vaccination, marital status, attitude towards immunization, occupational status (employment), place of delivery, parity, age, sex, religion, ethnicity, and residence.

### Operational definitions

**Missed opportunities for Vaccination**: A missed opportunity for vaccination (MOV) refers to any contact with health services by an individual (child or person of any age) who is eligible for vaccination (e.g., unvaccinated or partially vaccinated and free of contraindications to vaccination), which does not result in the person receiving one or more of the vaccine doses for which he or she is eligible (3–5).

**Eligible child:** A child whose age is 0 to 11 months and who needs immunization without any contradiction.

**Fully immunized:** A child who had completed vaccination against eight EPI-targeted diseases according to the standard vaccination schedule of the Ministry of Health of Ethiopia (1).

**Perception of caretakers**: The perception of caretakers was measured by comparing mean score responses to seven perception-assessing parameters (immunization is beneficial, childhood vaccines are safe, mothers should take their children for immunization, immunizations are provided free of charge, immunization can cause infertility later in life, the government promotes immunization for selfish interest, and local preparation can serve as a substitute for immunization). Those with scores at or below the mean were classified as having “no or poor perception” while those with scores above the mean were categorized as having “yes or good perception” (18).

### Data collection procedure and tools

A structured questionnaire was developed following a thorough review of the literature and a consideration of the local situation. It was first written in English and then translated into Amharic, the local language. Data were gathered through face-to-face interviews with a pre-tested and structured questionnaire. Two midwives with diploma degrees were used as data collectors, one degree midwives serving as supervisor.

### Data quality controls

Pre-testing was done, technical training was given to data collectors and supervisors, and a data collection tool was created after a review of the pertinent literature. On a daily basis, the supervisors and principal investigators checked the data for completeness, accuracy, and clarity. Then the necessary correction was made according to the aims of the study. The necessary correction was made in accordance with the study's objectives. Throughout the period of data collection, the principal investigator and supervisor conducted daily ongoing checks to ensure the accuracy of the data.

### Data processing, analysis, and presentation

Data were cleaned, coded, and entered into the Epidata version 4.0 before being transferred to SPSS version 20 for analysis. To summarize the data, a descriptive analysis was performed. A binary logistic regression analysis was performed to determine the association between independent and outcome variables. All predictor variables with *p* ≤ 0.2 were entered into multivariable logistic regression analysis; a significant association based on *p* ≤ 0.05 and an adjusted odd ratio (AOR) with 95% CI were identified. The results are presented in the form of texts and tables.

## Results

### Socio-Demographic characteristics of participants

Three hundred and forty-six children aged 0 to 11 months, who visited a health facility during the study period, were included, with a 100% response rate. Out of 346 children, 115 (33.2%) were younger than 45 days and 182 (56.6%) were between 45 days and 9 months. There were 194 (56.1%) male children and 152 (43.9%) female children among the included infants. Three hundred and twenty-seven (94.5%) of the babies were born in hospitals. The purpose of children's visits to the health facility was for treatment of sickness 129 (37.3%), vaccination 174 (50.3%), and other purposes 43 (12.4%). Concerning the age of the caregiver, 26 (7.5%) were under the age of 18, 183 (52.9%) were between the ages of 18 and 35, and 137 (39.1%) were over 35. Regarding the educational status of caregivers, 129 (37.3%) had no formal education, 112 (15.3%) completed primary education, 53 (15.3%) completed secondary education, and the remaining 52 (15.0%) completed college level or higher. Approximately 206 (59.5%) caregivers were from urban areas, and the remaining 140 (40.5%) were from rural catchment areas. In terms of religion, there were 314 orthodox Christians (90.8%), 26 Muslims (7.5%), and 6 Protestants (1.7%) (see Table 1).

**Table 1:** Socio-demographic related characteristics of the respondents for missed opportunities for vaccination, 2022.

Variables	Category	Frequency	Percentage (%)	*X*^2^ square, df, *p*-value
Maternal age	<18	26	7.5	*X*^2 ^= 2.269 df = 2 *p*-value = 0.412
	18–35	183	52.9	
	>35	137	39.1	
Marital status	Single	9	2.6	*X*^2 ^= 2.305 df = 3 *p*-value = 0.316
	Married	302	87.3	
	Divorce	28	8.1	
	Widowed	7	2.0	
Maternal education	Unable to read and write	129	37.3	*X*^2 ^= 9.883 df = 3 *p*-value = 0.063
	Primary education	112	32.4	
	Secondary education	53	15.3	
	College and above	52	15.0	
Maternal occupation	Housewife	56	16.2	*X*^2^= 18.452 df = 3 *p*-value = 0.641
	Civil servant	46	13.3	
	Farmer	197	57.0	
	Merchant	47	13.6	
Religion	Protestant	6	1.7	*X*^2 ^= 1.305 df = 2 *p*-value = 0.316
	Orthodox	314	90.8	
	Muslim	26	7.5	
Residence	Urban	140	40.5	*X*^2 ^= 21.119 df = 1 *p*-value = 0.003
	Rural	206	59.5	
Age of infants (in months)	<45 days	115	33.2	*X*^2 ^= 6.177 df = 3 *p*-value = 0.613
	45days to 9month	182	56.6	
	9 to 11months	49	14.2	
Sex of infant	Male	194	56.1	*X*^2 ^= 9.883 df = 1 *p*-value = 0.407
	Female	152	43.9	
Place of delivery	Home	19	5.5	*X*^2 ^= 2.305 df = 1 *p*-value = 0.316
	Healthy facility	327	94.5	
Reason for visit	The child is sick	129	37.3	*X*^2 ^= 3.305 df = 2 *p*-value = 0.346
	Vaccination	174	50.3	
	Others	43	12.4	

### Knowledge and perception of caretakers on immunization

Regarding vaccine-preventable diseases, the assessment of caretakers' knowledge revealed that 95 (27.5%) of them were unaware of any of the ten vaccine-preventable diseases, while 165 (46.7%) of them knew about one to two vaccine-preventable diseases and 25 (7.2%) knew about six to nine vaccine-preventable diseases. Two hundred and thirty-seven (68.5%) caregivers indicated that the goal of vaccination is to prevent diseases, 67 (19.4%) of them said that children grow up healthy, and 133 (9.5%) of them said that vaccination can treat diseases (see Table 2).

**Table 2:** Knowledge and perception of caretakers for missed opportunities for vaccination, 2022.

Variables	Category	Frequency	Percentage	*X*^2^ square, df, *p*-value
Vaccination message in last month	Yes	103	29.8	*X*^2 ^= 1.567 df = 1 *p*-value = 0.903
	No	243	70.2	
Source of message for vaccination in last month	Media	85	24.6	*X*^2 ^= 0.305 df = 1 *p*-value = 0.967
	Health extension workers	18	5.2	
Previous vaccination history	Yes	331	95.7	*X*^2 ^= 0.119 df = 1 *p*-value = 0.730
	No	15	4.3	
Vaccination requested and refused by the provider	Yes	64	18.5	*X*^2 ^= 0.536 df = 1 *p*-value = 0.464
	No	282	81.5	
Reason of refusal	Sickness	3	0.9	*X*^2 ^= 0.481 df = 3 *p*-value = 0.523
	No vaccination supplies on that day	11	3.2	
	Was not vaccination day	42	12.1	
	The vaccination site was closed	8	2.3	
Knowledge of the number of schedules left/contact time	Yes	92	26.6	*X*^2 ^= 0.88 df = 1 *p*-value = 0.346
	No	254	73.4	
Caretakers knew the vaccine-preventable disease	Knows one to two diseases	165	47.7	*X*^2 ^= 1.29 df = 3 *p*-value = 0.256
	Knows three to five diseases	61	17.6	
	Knows six to nine diseases	25	7.2	
	Do not know	95	27.5	
Purpose of vaccination	To prevent disease	237	68.5	*X*^2 ^= 0.013 df = 1 *p*-value = 0.910
	To help children grow up to be healthy	67	94.4	
	To cure disease	33	9.5	
	Not sure what they are for	9	2.6	
Caretakers thought that there would be disease development if the child is not vaccinated	Yes	312	90.2	*X*^2 ^= 1.851 df = 2 *p*-value = 0.396
	No	17	4.9	
	Do not know	17	4.9	
Perception of caretaker	Yes	152	43.9	*X*^2 ^= 21.6 df = 1 *p*-value = 0.001
	No	17	4.9	

### The magnitude of missed opportunities for immunization

Among all study participants, the magnitude of the missed opportunity for immunization among infants was 49.1% with a 95% CI (43.7 to 52.9%). Major vaccines with a high missed opportunity were OPV 0 (35%), BCG (37.9%), and measles (31.1%), and the major reason for immunization for missed vaccines was due to the absence of an adequate number of children to conduct immunization sessions, which was approximately 81.2%.

### Factors associated with missed opportunities for immunization

Age of infants, age of caretaker, maternal education, residence, stock out of vaccine session, vaccination message, knowledge contact time, perception, and purpose of the visit were found to be candidate variables for multivariable logistic regression analysis.

Multivariable logistic regression analysis revealed that residence, maternal education, and perception about immunization were associated with missed opportunities for immunization at *p*-values ≤ 0.05.

Mothers who resided in rural areas were 4.32 times more likely to have missed opportunities than their counterparts (AOR = 4.32, 95% CI: 3.11, 6.38).

Mothers who are unable to read or write are twice as likely than their counterparts to miss immunization opportunities (AOR = 2.45, 95% CI: 2.14, 4.22).

Maternal perception towards immunization is another factor that affects missed opportunities. Mothers who had negative perceptions were two times more likely to have missed opportunities for immunization when compared to their counterparts (AOR = 2.13, 95% CI: 1.89, 4.07) (see Table 3).

**Table 3:** Multivariable analysis for factors associated with missed opportunities for vaccination, 2022.

Variables		Immunization status		COR(95% CI)	AOR(95% CI)
		Missed	Not missed		
Age of infants	<45 days	87	26	12.129 (0.114–0.637)	7.15 (0.61, 31.54)
	45 days to 9 months	26	56	0.284 (0.162–0.499)	0.169 (0.044, 2.655)
	9 to 11 months	28	21	1	1
Age of caretaker (yr.)	< 18	3	12	1	1
	18 to 35	50	67	1.348 (1.061–3.941)	0.83 (0.03, 18.31)
	>35	14	6	0.421 (0.609–28.303)	0.51 (0.02, 13.38)
Maternal education	Unable to read and write	60	69	9.55 (4.73–19.26)	**2.45** (2.14, 4.22)*
	Able to read and write	64	48	4.29 (1.92–9.58)	3.39 (0.12, 1.91)
	Primary education	26	27	0.44 (0.05–3.62)	0.56 (0.71, 1.88)
	Above Secondary	20	32	1	1
Residence	Rural	40	25	1.86 (2.58–10.24)	**4.32** (3.11, 6.38)*
	Urban	130	151	1	1
Vaccination message	No	131	112	1.92 (0.26–4.71)	0.37 (0.11,1.32)
	Yes	39	64	1	1
Knowledge contact time	No	139	115	1.19 (0.63–7.32)	0.36 (0.15,0.86)
	Yes	94	93	1	1
Perception of caretakers	No	17	6	2.98 (1.95–6.57)	**2.13** (1.89, 4.07)*
	Yes	152	160	1	1
Purpose of visit	The child is sick	87	26	0.413 (0.351–6.921)	0.32 (0.046,3.82)
	Vaccination	26	56	1.213 (0.647–9.812)	1.39 (0.28,6.83)
	Others	28	21	1	1

1 = reference category (infant age between 9 and 11 months, age of caretaker < 18 years, maternal education attended greater than secondary school, urban residence, source of vaccination message, having knowledge of contact time of vaccine, have a good perception of caretakers and others (children to grow up healthy, not sure what they are for).

* Statistically significant.

## Discussion

The purpose of this research was to find out how common missed opportunities for immunization are and what factors contribute to them in the study area. In this research, the proportion of missed opportunities for immunization was 49.1%. The findings were consistent with vaccine assessments missed in Chad (51%) (19) and Gurage zone, Ethiopia (49.1%) (20). This could be due to similarities in the nature of the study, the study design, the study population, and the healthcare infrastructure.

This study is significantly greater than the cross-sectional research conducted in the Sidama zone (28.8%) (14), Jimma hospital (28.8%) (17), Ambo, central Ethiopia (23.7%) (16), Kenya (16.2%) (21), Timor Leste (41%) (22), Nigeria (32.8%) (23), and South Africa (14.1) (24). However, the finding is also less than the research done in the East Gojjam Zone, Ethiopia (74.9%), South Sudan (56.5%), Mozambique (76%), Malawi (66%), Kenya (75%), and Burkina Faso (76%) (15, 19, 25–27). This might be a result of a lack of infrastructure for access to healthcare facilities. Furthermore, this finding might also differ from others due to socioeconomics, study area, and study period differences.

Additionally, we attempted to evaluate factors that might contribute to missed opportunities for immunization. The results show that maternal educational status, place of residence, and caregivers’ attitudes toward the vaccine all had statistically significant correlations with missed opportunities for immunization among infants in the age range of 0 to 11 months.

Children born to mothers whose level of education is low were more likely to be unvaccinated compared with their counterparts. It was supported by research done in the Ilorin metropolis (28), South Africa (24), Mozambique (29), and the East Gojjam Zone, Ethiopia (25). The possible reason for more missed opportunities could be a lack of knowledge about the benefits of vaccines for their children. On the other hand, the level of education itself affects access to immunization messages since they could not read reliable information and even appointment dates from cards. However, maternal education is not consistently associated with missed opportunities for immunization. This is similar to the research done in Saudi Arabia (24).

Place of residence was another factor that affected the use of immunization among infants. We found that children who lived in rural areas were more likely to miss opportunities for immunization compared with urban ones. This is consistent with the findings of the study conducted in Nigeria, which revealed that long-distance walking was the major reason for missed opportunities for the vaccine (30). This could be due to the accessibility of the vaccine location, which could be the cause of more missed opportunities for immunization.

The proportion of missed opportunities for immunization was associated with caregiver perceptions. Caregivers with a negative perception about the about the side effects of vaccines is twice more likely to have for missed opportunities for immunization compared to their counterparts. This is similar to previous studies done in southern Ethiopia and Uganda (14, 31). The possible explanation could be that mothers who are concerned about vaccine side effects either refuse or postpone immunizations.

The limitation of this study was its lack of generalizability due to its single setting and institutional basis. Recall biases are another limitation of this study. In this study, we did not use a standard for measuring missed opportunities for immunization, and we used a Qualitative approach that could not address the “why” questions in detail.

## Conclusion

The proportion of missed opportunities for immunization was 49.1%, which is high. Low level of education, rural residence, and perception of caretakers were factors affecting missed opportunities for immunization. There should be a system in place to provide mothers and caregivers with information and education about immunization services for their children.

## Data Availability

The original contributions presented in the study are included in the article, further inquiries can be directed to the corresponding author.
